# Forebrain development in the *Inpp5e* ciliary mouse mutant is severely disrupted

**DOI:** 10.1186/2046-2530-1-S1-P106

**Published:** 2012-11-16

**Authors:** T Theil, K Hasenpusch-Theil, D Magnani, S Gayral, S Schurmans

**Affiliations:** 1 University of Edinburgh, UK; 2 Université de Liège, Belgium

## 

Ciliopathies are an emerging group of related genetic disorders caused by defects in the function and/or structure of primary cilia, antenna like extensions protruding from the cell surface with important functions in signalling and sensory perception. Ciliopathies are characterized by pleiotropic clinical features and almost every organ in the body can be affected. Notably, many ciliopathy patients display important neurological features, most commonly mental retardation (MR), however, little is known about the pathogenesis underlying MR. Mutations in human *INPP5E* have been identified in MORM syndrome and Joubert syndrome (JS). JS is a genetically and phenotypically heterogeneous syndrome which can lead to MR and can also be associated with autism spectrum disorder. Here, we present the analyses of forebrain development in the *Inpp5e* ciliary mouse mutant. We show that in newborn *Inpp5e* mutants the corpus callosum is hypoplastic, consistent with callosal malformations often observed in ciliopathy patients. Also, the thalamocortical tract shows severe pathfinding defects. Many thalamocortical axons (TCAs) take an abnormal trajectory into the cortex while other TCAs are deflected ventrally towards the amygdala. In addition, while all hippocampal fields are specified, hippocampal size is severely reduced coinciding with a down-regulation of Wnt/βcatenin signalling during patterning stages. Finally, the cerebral cortex is extremely thin at caudal levels and exhibits cortical heterotopias with a local outgrowth of cortical tissue. Taken together, these findings indicate severe forebrain malformations in *Inpp5e* mutants offering a rare opportunity to study the pathogenesis of MR in a JS mouse model.

